# Social signal processing for studying parent–infant interaction

**DOI:** 10.3389/fpsyg.2014.01437

**Published:** 2014-12-10

**Authors:** Marie Avril, Chloë Leclère, Sylvie Viaux, Stéphane Michelet, Catherine Achard, Sylvain Missonnier, Miri Keren, David Cohen, Mohamed Chetouani

**Affiliations:** ^1^CNRS, Institut des Systèmes Intelligents et de Robotiques, UMR 7222, Université Pierre et Marie CurieParis, France; ^2^Department of Child and Adolescent Psychiatry, Pitié-Salpêtrière HospitalParis, France; ^3^Laboratoire de Psychologie Clinique et Psychopathologie, Psychanalyse, Paris René Descartes UniversityBoulogne, France; ^4^Department of Psychiatry, Infant Mental Health Unit, Geha Hospital, Tel Aviv UniversityTel Aviv, Israel

**Keywords:** early parent–infant interaction, feature extraction, multimodal computational analysis, RGB-D sensor, synchrony, social signal processing

## Abstract

Studying early interactions is a core issue of infant development and psychopathology. Automatic social signal processing theoretically offers the possibility to extract and analyze communication by taking an integrative perspective, considering the multimodal nature and dynamics of behaviors (including synchrony). This paper proposes an explorative method to acquire and extract relevant social signals from a naturalistic early parent–infant interaction. An experimental setup is proposed based on both clinical and technical requirements. We extracted various cues from body postures and speech productions of partners using the IMI2S (Interaction, Multimodal Integration, and Social Signal) Framework. Preliminary clinical and computational results are reported for two dyads (one pathological in a situation of severe emotional neglect and one normal control) as an illustration of our cross-disciplinary protocol. The results from both clinical and computational analyzes highlight similar differences: the pathological dyad shows dyssynchronic interaction led by the infant whereas the control dyad shows synchronic interaction and a smooth interactive dialog. The results suggest that the current method might be promising for future studies.

## INTRODUCTION

Parent–child interactions are crucial for learning, later psychological traits, and psychopathology (Cohen, 2012). In many species, including mammals, parent–child interactions are based on close relationships that are characterized by (i) infant dependency on caregivers and (ii) a specific communication dynamic associated with a caregiver’s adaptation and infant maturation. However, this type of study is complex, requiring the perception and integration of multimodal social signals. Combining several approaches within a multidisciplinary perspective at the intersection of social signal processing, computational neuroscience, developmental psychology, and child psychiatry may efficiently investigate the meaning of social signals during early parent–child interaction (Meltzoff et al., 2009). Exploring normal and pathological interactions during this early period of life has many implications including the possibility of understanding what the baby partner cannot explicitly express due to immaturity.

The Syned-Psy project (Synchrony, Early Development and Psychopathology, http://synedpsy.isir.upmc.fr/) aims to improve the synergy among three fields: child psychiatry, developmental psychology and social signal processing. The idea is to understand the clinical relevance of synchronic and dyssynchronic dyadic interactions and to develop automatic algorithmic tools to detect these phenomena in natural settings. Originally conceptualized and studied by developmental psychologists, the concept of synchrony is now relevant to many different research fields including social signal processing, robotics and machine learning. According to its conceptual framework, synchrony can be defined in many ways (Leclère et al., 2014). Delaherche et al. (2012) recently proposed that in most cases, one should distinguish between *what* is assessed (i.e., modalities such as body movement, gaze, smile, and emotion) and *how* the temporal link between partners’ different modalities of interaction are assessed (i.e., speed, simultaneity, and smoothness). In the rest of the manuscript, we will follow this definition of synchrony.

The aim of this work was to characterize synchrony/dyssynchrony in parent–infant interactions occurring in situations of severe emotional neglect and to select interaction metrics that may be used in future clinical trials. To do this, we proposed to automatically detect and analyze behaviors. These behaviors are selected by considering clinical and technical requirements. Furthermore, the objective of our approach was to explore the capacity of new technological devices and tools to understand early parent–child interactions.

### RELATED WORK IN PSYCHOLOGY

The quality of the parent–child relationship impacts children’s social, emotional and cognitive development (Harrist and Waugh, 2002; Saint-Georges et al., 2013). Describing parent–child behavioral interactions is not a simple task because there are multiple modalities of interaction to explore. First, the interactive partnership between an infant and caregiver (usually called a “dyad”) has to be defined and explored as a single unit. Second, given that the relationship between an infant and their caregiver is bidirectional in nature, the dyad should be thought of as a dynamically interacting system (Sameroff, 2009). Third, given the dynamic relationship between an infant and their caregiver, a specific interest in the flow characterizing the exchange of information during infant-caregiver interactions has emerged (Weisman et al., 2012, 2013), leading to the study of rhythm (Berry et al., 1974; Condon, 1986; Stern, 2009), reciprocity (Lebovici, 1985; Bråten, 1998), and synchrony (Feldman, 2007). The recent discovery of both biological correlates of behaviorally synchronic phenomena (Dumas et al., 2010) and statistical learning (Kuhl, 2003; Saffran, 2003) has validated the crucial value of studying synchrony during child development (Feldman, 2007; Cohen, 2012). It appears that synchrony should be regarded as a social signal *per se* as it has been shown to be valid in both normal and pathological populations. Better mother–child synchrony is associated with familiarity (vs. unknown partner), a healthy mother (vs. pathological mother), typical development (vs. psychopathological development), and a more positive child outcome (Leclère et al., 2014).

In the field of human interactions, interactional synchrony can be defined as “the dynamic and reciprocal adaptation of the temporal structure of behaviors between interactive partners” (Delaherche et al., 2012). Here, behaviors include verbal and non-verbal communicative and emotional behaviors (e.g., gestures, postures, facial displays, vocalizations, and gazes). Synchronous interactions entail coordination between partners and intermodality. Caregivers and their children are able to respond to each other using different modalities starting from birth (Vandenberg, 2006; Hart, 2010). Thus, synchrony differs from mirroring or the chameleon effect. Instead, synchrony describes the intricate “dance” that occurs during short, intense, playful interactions; it builds on familiarity with the partner’s behavioral repertoire and interaction rhythms, and it depicts the underlying temporal structure of highly aroused moments of interpersonal exchange that are clearly separated from the stream of daily life (Beebe and Lachmann, 1988; Tronick and Cohn, 1989; Fogel et al., 1992; Bråten, 1998; Stern, 2009). Therefore, synchrony has been measured in many different ways due to its broad range of theoretical applicability. The most common terms referring to synchrony are mutuality, reciprocity, rhythmicity, harmonious interaction, turn-taking and shared affect; all terms are used to characterize the mother–child dyad. Three main types of assessment methods for studying synchrony have emerged: (1) global interaction scales with dyadic items; (2) specific synchrony scales; and (3) micro-coded time-series analyzes (for a detailed review, see Leclère et al., 2014).

### RELATED WORK IN COMPUTATIONAL PROCESSING

Many studies have been conducted (Gatica-Perez, 2009) to assess social interactions using automatic and computational methods, including automatic extraction of non-verbal cues and/or models of the multimodal nature of interaction. These studies have been performed in various contextual applications including role recognition (Salamin et al., 2009), partner coordination during interaction (Hung and Gatica-Perez, 2010), automatic analysis of meeting (Campbell, 2009; Vinciarelli et al., 2009), studying interactive virtual agents (Prepin and Pelachaud, 2011), and understanding of early development (Meltzoff et al., 2009). In the health domain, these applications include recognition or classification of psychopathological states (Cohn, 2010), psychotherapeutic alliance (Ramseyer and Tschacher, 2011), classification of autistic dimensions (Demouy et al., 2011) or the recognition of early expression of autism (Cohen et al., 2013).

Signals that have been investigated during social interactions are specific because they are not semantic in nature and often occur without consciousness. They include amplitude, frequency and duration for the non-verbal signals such as fillers, backchannels or gestures. Vinciarelli et al. (2009) distinguish five categories of cues: (1) physical appearance; (2) gesture and posture; (3) gaze and facial behaviors and mimics; (4) vocal cues; and (5) behavior related to the space and environment. Regarding audio signals, some cues have been better studied such as pitch, intensity and vocal quality (Batliner et al., 2011), intonation (Ringeval et al., 2011), rhythm (Hogan, 2011), motherese (Saint-Georges et al., 2013), and perceived emotion (Schuller et al., 2010). Regarding video signals, cues usually investigated include the quantity of body movements (Altmann, 2011; Ramseyer and Tschacher, 2011; Paxton and Dale, 2014) or facial movements (Carletta et al., 2006), the study of hand movements (Marcos-Ramiro et al., 2013; Ramanathan et al., 2013) or finger movements (Dong et al., 2013), the study of gaze (Sanchez-Cortes et al., 2013), and data with a higher level of annotation including smiling (Rehg et al., 2013), facial expressions (Bilakhia et al., 2013), posture (Feese et al., 2012) or the emotional body language (McColl and Nejat, 2014). In the era of RGB-D sensors (e.g., Kinect), online extraction of the skeleton is now available and has enabled the study of action recognition based on the joint architecture of the human body (Aggarwal and Xia, 2014; Chan-Hon-Tong et al., 2014). As a consequence, new body movement cues have been proposed based on the position of articulated arms, the trunk, head, and legs (Caridakis and Karpouzis, 2011; Yun et al., 2012; Anzalone et al., 2014a).

Some cues have been extracted to assess social characteristics and interaction at the level of the dyad (Yun et al., 2012; Ramanathan et al., 2013). Several studies (Campbell, 2009; Delaherche et al., 2012; Bilakhia et al., 2013; Rolf and Asada, 2014) have considered the multimodal nature of social signals and simultaneously studied several modalities. Various authors have used different metrics and modeling techniques to study synchrony (Delaherche et al., 2012), including correlation (Altmann, 2011), recurrent analysis (Varni et al., 2009), regression models (Bilakhia et al., 2013), quantity of mutual information (Rolf and Asada, 2014), or influence models (Dong et al., 2013).

### PAPER CONTRIBUTION AND ORGANIZATION

The aim of this paper is to describe our methodology and to test its feasibility. Here, we present a pilot study in which we extracted and analyzed behavioral features in two case reports, one pathological situation of severe emotional neglect and one normal control, to study the feasibility and the coherence of the method. From an experimental point of view, the particularity of this work is to employ a computational setup in a clinical setting, where both needs and constraints had to be completed. The acquisition application had to preserve a natural free-play interaction between pathological dyads and be usable by a non-expert. All the interactive scenarios and the applications have been designed in collaboration with psychologists. This collaboration has continued with the selection of relevant behavioral features from the raw data and their interpretation. The rest of the paper is organized as follows: in section 2, we present the method used to set up a computational system in a clinical setting and how we analyzed data acquired during the interactions. In section 3, results of clinical and computational analysis are presented for two representative dyads, and in section 4, the method and results are discussed.

## MATERIALS AND METHODS

In this section, we focus on the integration of a computational setup in a clinical study and how the data recorded during the interaction can be treated. From a clinical point of view, the protocol aims to offer an optimal acquisition of parent–infant interactions and to preserve the natural interaction. The method of acquiring data must be as minimally intrusive as possible. From a technical perspective, the acquisition must be sufficiently efficient and robust to be able to collect significant and exploitable data for off-line processing.

### CLINICAL PROTOCOL

The current protocol is part of a clinical study conducted in a French perinatal ambulatory unit “Unité Petite Enfance et Parentalité Vivaldi” of the Pitié-Salpêtrière University Hospital. The main objective of the study, named “*ESPOIR Bébé Famille*,” is to evaluate the relevance of an early intensive intervention program for dyads in severe child neglect (CN) situations. CN is the persistent failure of the caregiver to meet the child’s basic physical and/or psychological needs, resulting in interaction disorders (Glaser, 2002) and serious impairment of the child’s development with short and long term negative impacts on the child’s cognitive, socio emotional, behavioral and psychological development and emotional regulation (Rees, 2008). Thus, a severe neglectful situation presents interaction difficulties and dyssynchrony.

The inclusion criteria were as follows: (1) Dyads consisted of mothers (or fathers) with their children whose age varied between 12 and 36 months. At 12 months, the interactive pattern of the dyad is already built, and data extraction is facilitated because the child is able to sit in a small chair. The oldest age accepted was 36 months because that is the age limit for the parent child health care in this unit. (2) Mothers (or fathers) have been referred to the unit by social services or court petitions due to CN. (3) Clinical confirmation of CN is based on a child psychiatrist’s assessment using the PIRGAS scale (Parent–Infant Relationship Global Assessment Scale, Axe II of DC 0-3 R), a clinical intensive scale of parent–child interaction quality. A control group of dyads with normal development and without interactional difficulty was also recruited.

The clinical evaluation of these dyads included interviews, questionnaires and filmed play sessions used for clinical annotations. Specifically, to assess synchrony, we used the coding interactive behavior (CIB), which is one of the most often used and validated global interaction scales (for a review of clinical instruments see Leclère et al., 2014). The CIB includes 43 codes rated on a 5-point Likert scale, divided into parent, child and dyadic codes. Codes were averaged into eight composites that were theoretically derived, concerned with diverse aspects of early parent–infant relationships and showed acceptable to high levels of internal consistency (Feldman et al., 1996; Keren et al., 2001). The French version has been validated and offers the same factorial distribution (Viaux-Savelon et al., 2014). The composites and items used in the present study are presented in **Table 1**.

**Table 1:** CIB relative items according to the eight composite subscores.

Composites	Relatives items
Parental sensitivity	Acknowledging; imitating; elaborating; parent gaze; positive affect; vocal appropriateness, clarity; appropriate range of affect; resourcefulness; praising; affectionate touch; supportive presence; infant led interaction
Parent intrusiveness	Forcing-physical manipulation; overriding, intrusiveness; parent negative affect, anger; parent anxiety; criticizing; parent-led interaction
Parent limit setting	Consistency of style; resourcefulness; appropriate structure, limit setting
Child compliance	Compliance to parent; reliance on parent for help; on-task persistence
Child withdrawal	Child negative emotionality, fussy; withdrawal; labile affect; avoidance of parent
Child engagement	Joint attention; child positive affect; affection to parent; alertness; fatigue; vocalizations, verbal output; initiation; competent use of the environment; creative-symbolic play; infant-led interaction
Dyadic joint negative state	Parent negative affect, anger; hostility; child negative emotionality, fussy; withdrawal, labile affect; fatigue; constriction; tension
Dyadic reciprocity	Parent gaze; positive affect; praising; affectionate touch; joint attention; child positive affect; vocalization, verbal output; initiation; dyadic reciprocity; adaptation-regulation; fluency

The proposed computational system has been used in the filmed play sessions where parents and infants have a natural interaction. Play session are composed of three stages to capture the dyad behaviors in different contexts: (1) Free interactive play (4 min): parent and infant are invited to play together with toys as usual. The goal is to create an interaction that is as natural as possible; the only directive given is “play as if you were at home.” (2) Directed game (2 min): a complex game is given to the child (a puzzle for example) to encourage the parent to help them. With a difficult game, the purpose is to determine how the child will solicit the parent and how the parent will respond. In addition, this situation will incite the parent to intervene spontaneously during the game. (3) Free play while the parent is occupied (2 min): a questionnaire is given to the parent while the child is playing with toys. In this final situation, the aim is to observe how the child solicits the parent and how the parent shares their attention between the task and their infant.

**FIGURE 1:**
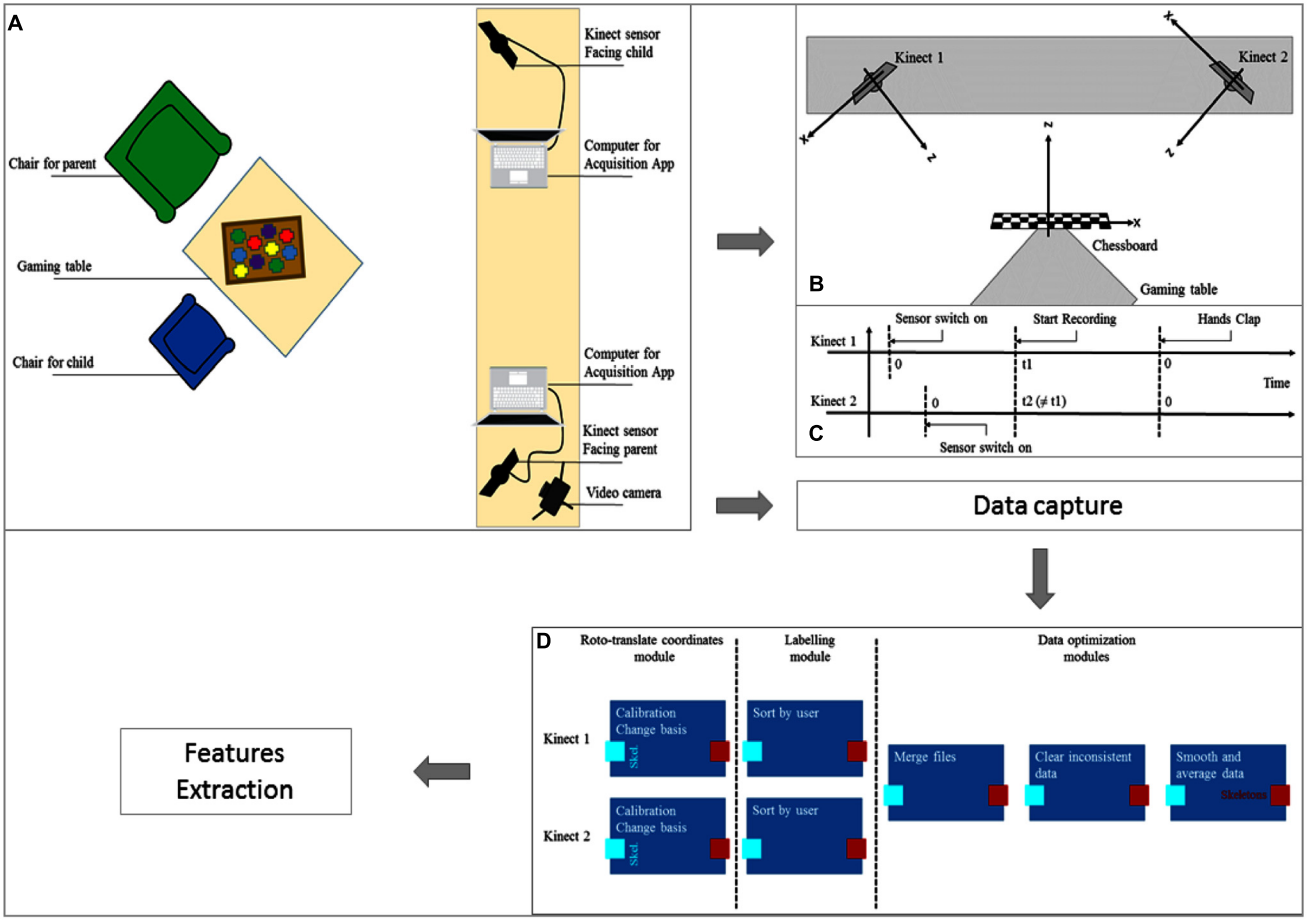
**Data recording and extraction. (A)** Play room and materials; **(B)** 3D-calibration with a chessboard; **(C)** Time synchronization with a hand clap; **(D)** Skeleton coordinates pre-processing pipeline.

Play sessions take place in a consultation room, controlled by a psychologist, where the parent and infant are invited to sit around a small table to play. Although a face-to-face disposition facilitates interactions, it complicates the data acquisition. Thus, the parent and infant are placed at 90° to one another around the table. To collect information from the interaction, two synchronized RGB-D sensors are placed in front of each participant and connected to a computer. This will run an acquisition application to record scene data. Additionally, a camera is used to film the scene for the clinical evaluation. **Figure 1A** shows the hardware setup in the consultation room.

Given our aim to run a study in a clinical setting, the acquisition application has to be easy to use and robust. Indeed, dyads with emotional neglect present interaction difficulties and thus the play sessions are subject to variations due to the child’s (e.g., standing on a chair, looking for other toys) and parent’s behavior (e.g., difficulty in controlling their child, wearing a large coat, hiding their face). Moreover, the psychologist has to leave the room each time the play session takes place. To reach these needs: (i) the hardware system is hidden to offer the most natural environment possible and avoid interest and distraction from the participants. (ii) The psychologist had to prepare the parent for the presence of a camera that is sometimes problematic. (iii) The hardware and the acquisition application were computed to be easily setup.

### ACQUISITION APPLICATION

To respond to all of the technical and clinical constraints cited above, an acquisition application has been implemented with a robust and efficient framework and the ability to collect the maximum amount of significant data while remaining easy to use by a non-professional.

As mentioned above, the scene is recorded by two Kinects, low-cost RGB-D sensors designed by Microsoft. These devices, mainly used for gesture recognition, offer the possibility to record many signals from a scene with only one device. The setup incorporates a color camera, depth sensor based on a structured light technique and a microphone array. Coupled with the Microsoft SDK for Kinect, the setup allows the user to directly extract color images, depth images and also 3D coordinates of the skeletons and faces of the participants from a scene in real time. In our case, participants still are too far from the Kinect, so face tracking features are not used. Moreover, as participants are seated, only the upper-body skeleton tracking is activated.

The two Kinects are optimally placed in front of each participant to capture as much information as possible. However, 3D coordinates are obtained in a Kinect centered basis, therefore, trackers record different positions for each sensor. Thus, a spatial calibration of the Kinects is necessary, which is performed by chessboard calibration; a chessboard is placed in the field of view of the Kinects (laid on the gaming table) while the Kinects record the 3D coordinates of significant points of the chessboard (corners of squares). **Figure 1B** shows the calibration step with axis representation. These coordinates will be used later to compute the roto-translation matrix between the two Kinects to transform 3D points tracked into the same spatial basis.

A temporal synchronization is also needed for the Kinects. The internal sensor’s clock starts when the device is connected to the computer. As it is impossible to start the two sensors at the exact same time, a temporal synchronization is performed from the microphone outputs. When the Kinects detect a powerful sound for the first time (applause), they record the current timestamp as the beginning of the recording (see **Figure 1C** for a graphical view of the timelines). Then, each Kinect will have the same detection times.

Data captured by the Kinects must be recorded for oﬄine processing. To avoid computer overload during the acquisition (and offer the most efficient recording rate), minimal online processing is performed, and the raw data are saved in a lightweight format. For each sensor, saved data include:

• Color stream in an .avi video file (XVID codec) + timestamp for each image in an .xml file
• Depth stream in an .avi video file (XVID codec) + timestamp for each image in an .xml file
• Audio stream in an audio file (.wav)
• Audio source angle in an .xml file
• Skeleton tracked points (position and orientation) in an .xml file
• 3D calibration data in an .xml file

To facilitate the use of the acquisition application by a non-expert user, a graphical interface has been added. The graphical interface is divided into two windows, one for the visualization of the Kinect stream and the other for parameter management. In the first window, the user can display the Kinect stream, start and stop the recording and also modify the sensor tilt. A message field to display current acquisition status is proposed. In the second window, the user can choose the path to save the recorded data, such as the name of the folder, if tracked skeletons are displayed, or the number of squares on the calibration chessboard. This interface simplifies the use of the acquisition application and allows the verification and correct execution of the recordings.

### COMPUTATIONAL ANALYSIS

To extract and analyze the recorded data during the game session, a lightweight framework developed by the IMI2S ISIR group is used (Anzalone et al., 2014b). This framework is a distributed computing software platform that copes with the high level of complexity by simplifying the functional decomposition of the problems through the implementation of highly decoupled, efficient, and portable software. The developers implemented complex solutions using simple, small, and basic operative units that are able to interact between each other. Such basic modules are executed as independent computational units able to solve a particular problem. Inputs and outputs of different modules are then connected to exploit the main, complex problem.

In this study, the IMI2S framework is used to divide records into three segments of data according to the three types of game sessions, preprocess 3D skeleton data and, eventually, to extract behavioral features.

#### Skeleton preprocessing

As previously described, the use of two RGBD-sensors requires a basis change to obtain 3D coordinates in the same Cartesian space. In addition, to retain a maximum amount of information, data from each sensor must be merged before any treatment. **Figure 1D** presents the pre-processing pipeline for skeleton data from the two displaced sensors. Skeleton data of the parent and child from both sensors are corrected to belong to the same Cartesian space; each skeleton is then labeled, identifying the two users in the scene, the parent and the child. Finally, the data are merged into a unique stream, inconsistent skeletons are suppressed (for example if the tracked skeleton is misplaced) and the data are smoothed through average filtering.

#### Skeleton processing

After smoothing and cleaning of the skeleton data, several features can be extracted with IMI2S Framework. With 3D coordinates of 10 significant body points for each participant in a unique basis, distances and orientation features can be computed. Many examples of relevant skeleton features will be presented in the section “Results.”

#### Speech processing

We focused on voice activity detection (VAD) estimated through the OpenSmile framework (Eyben et al., 2010). When this feature was combined with the IMI2S framework, we obtained the probability of VAD.

In addition, when the method used by Galatas et al. (2013) was combined with the skeleton localization in space, it was possible to determine an audio source in the 3D space of the clinical room. Consequently, if a sound was detected, it could be associated with a user, even though distinguishing voices from other sounds (moving a toy, moving chair, etc.) is not currently efficient.

### SELECTION OF RELEVANT FEATURES

We deliberately reduce the number of features using a consensus multidisciplinary approach to select the most relevant ones. This was done by going back and forth between engineers and psychologists. First, engineers listed a series of features available from skeleton and audio processing for each partner. Second psychologists discussed with engineers how combining each partner feature could be related to a relevant clinical dimension in terms of communication. We focused on features related to proximity, motor and audio activity, and attention to the task and/or to the partner (see Result). Finally, we determined together higher level features related to synchrony and engagement during the interaction with the aim of selecting a limited number of features for clinical assessment.

## RESULTS

The current results focus only on two case reports, one pathological dyad in a severe emotional neglect situation and one control dyad with no interaction difficulty. The pathological dyad is composed of a 25-year-old mother and her 35-month-old boy. The interaction quality is rated as a 45 on the PIRGAS scale (DC 0-3 R). The control dyad is composed of a 29-year-old father and his 19-month-old boy. The interaction quality is rated as a 95 on the PIRGAS scale.

The analyzes were performed for the first phase in the ESPOIR protocol, the free play, where the parent and child are invited to play as they would at home to create as natural of an interaction as possible in the experimental scenario. It should be noted that in these experiments, the psychologist was present in the room with the dyad and stood at the bench between the two computers (see **Figure 1A**). Thus, she was a possible point of attraction during the experiment.

We present successively (1) the clinical assessment; (2) features related to proximity and motor activity; (3) features related to attention to the task and/or to the partner; and (4) participation to the task. Please note that natural interaction does not allow us to extract behavioral features during the entire time of the video session. For instance, data are missing when the child moves from the chair and is off-camera or when he climbs on his parent’s knees. A blank or a cross line in figures indicates uncollected data. By convention, results concerning parents are in green, and results concerning children are in blue.

### BLIND ASSESSMENT OF THE INTERACTION WITH THE CIB

**FIGURE 2:**
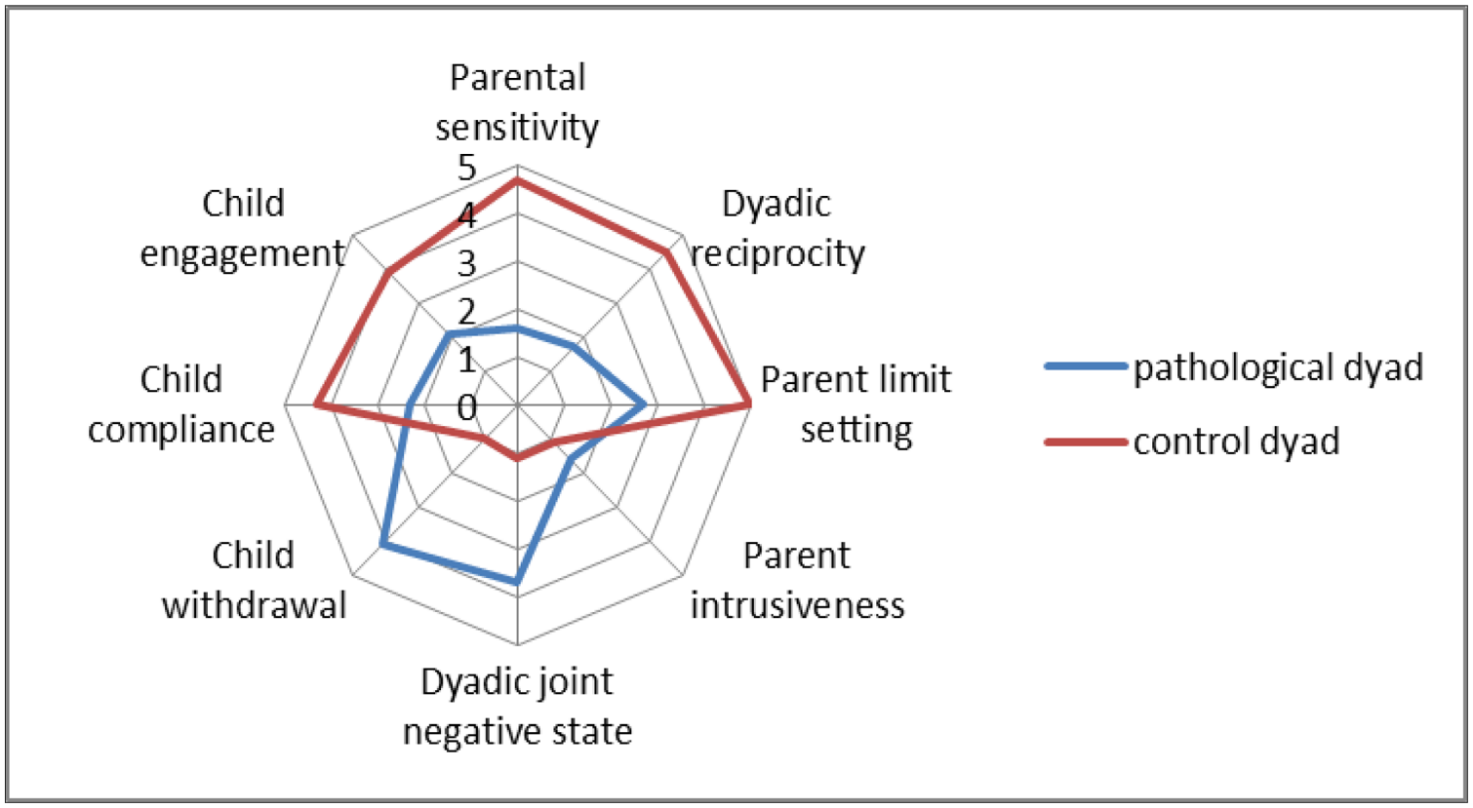
**Coding interactive behavior results for the pathological and control dyads**.

As expected (**Figure 2**), the control dyad presented significantly higher scores in the CIB positive domains (parental sensitivity, parent limit-setting, child compliance, child engagement, and dyadic reciprocity), and the pathological dyad presented higher scores in the negative domains (Dyadic joint negative state and Child withdrawal). The only domain showing a limited difference was Parent intrusiveness.

### PROXIMITY AND ACTIVITY FEATURES

**FIGURE 3:**
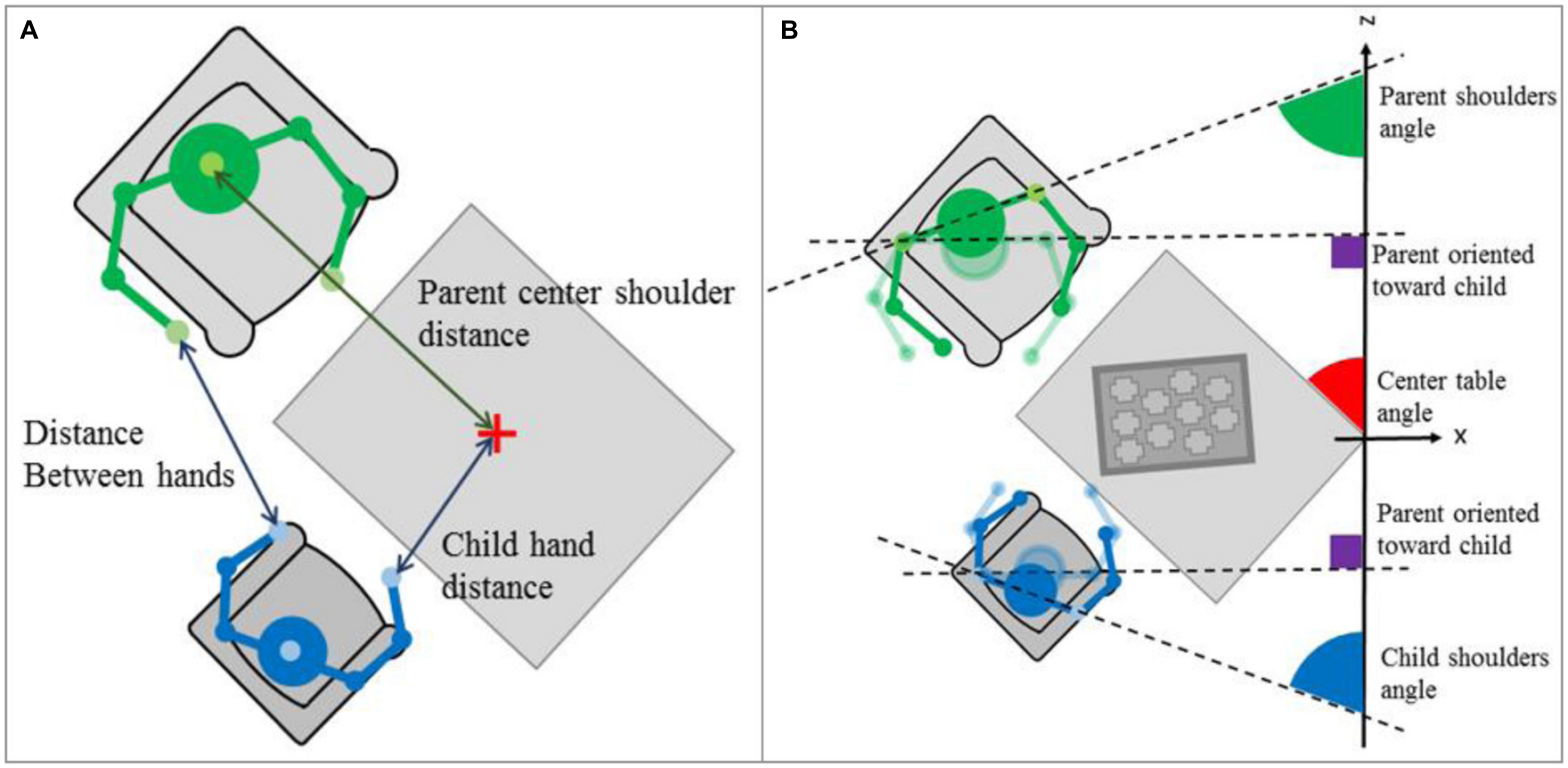
**(A)** Shoulder center and hand distance to the table center features; **(B)** Shoulder orientation feature – top view representations.

In this paragraph, we present low level features related to physical proximity during the task and motor activity. The main idea is to assess (1) how close partners are to one another and (2) how close partners are to the table where part of the interactions should occur. Several skeleton features have been developed in the IMI2S Framework to extract information concerning the proximity between the parent and child during the game session. Furthermore, these features reveal the general body activity of the participants. **Figure 3** offers a visual representation of (1) the distance between the shoulder center of a participant and the center of the gaming table. The shoulder center is the geometrical middle between the left and right shoulders. (2) The distance between each hand of the dyad (parent’s left hand-child’s right hand and parent’s right hand-child’s left hand).

**FIGURE 4:**
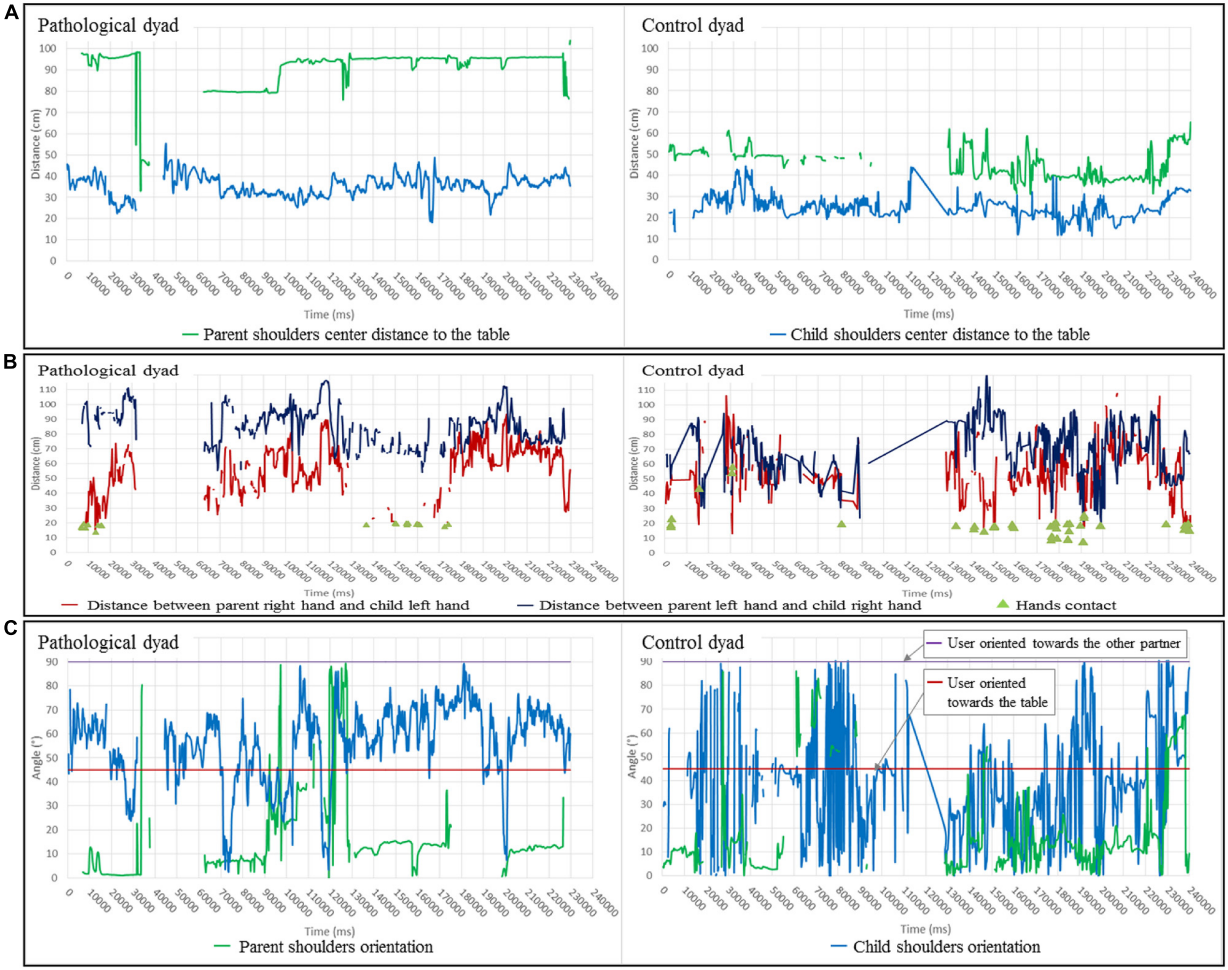
**(A)** Evolution of the distance between the shoulder center and the table center during the interaction. **(B)** Evolution of the distance between the parent’s and child’s hands during the interaction. **(C)** Evolution of shoulder orientation during the interaction (Left: pathological dyad; Right: control dyad). A blank or a cross line in figures indicates uncollected data. By convention, results concerning parents are in green, and results concerning children are in blue **(A,C)**.

The results are presented in **Figures 4A,B**, respectively. The pathological dyad is on the left, whereas the control dyad is on the right. **Table 2** summarizes the conclusions for these features.

**Table 2:** Proximity and body activity features – results analysis.

	Pathological dyad	Control dyad
Shoulder center distance to the table center	Mother far from the table (average = 80 cm) and is not moving Child near the table (average = 40 cm)	Parent near the table (average = 50 cm)
Distance between hands (please note that partners’ asymmetry is also a consequence of the seating position)	Few hand contacts (*N* = 20). Hands are far and distances between parent’s left hand-child’s right hand (average = 80 cm) and parent’s right hand-child’s left hand (average = 40 cm) represent partners’ asymmetry during the task	Not many hand contacts (*N* = 25). Hands are closer, and more importantly, distances between parent’s left hand-child’s right hand (average = 60 cm) and parent’s right hand-child’s left hand (average = 50 cm) break partners’ asymmetry

Conclusion	Pathological parent moves less and stays farther from their child than the control parent. Control dyad seems to interact more closely

#### Shoulder orientation results

To determine the torso orientation of a person, the angle between the line formed by the two shoulder points tracked and the line of the z axis has been computed (see **Figure 3B** for a graphical representation). In the current situation, if the person is oriented toward the gaming table, the formed angle will be ∼45° (red line in graphs). Moreover, if the person looks at their partner’s spot, the angle will be ∼90° (purple line in graphs). **Figure 4C** displays shoulder orientation for the two dyads.

#### Relative shoulder orientation results

**FIGURE 5:**
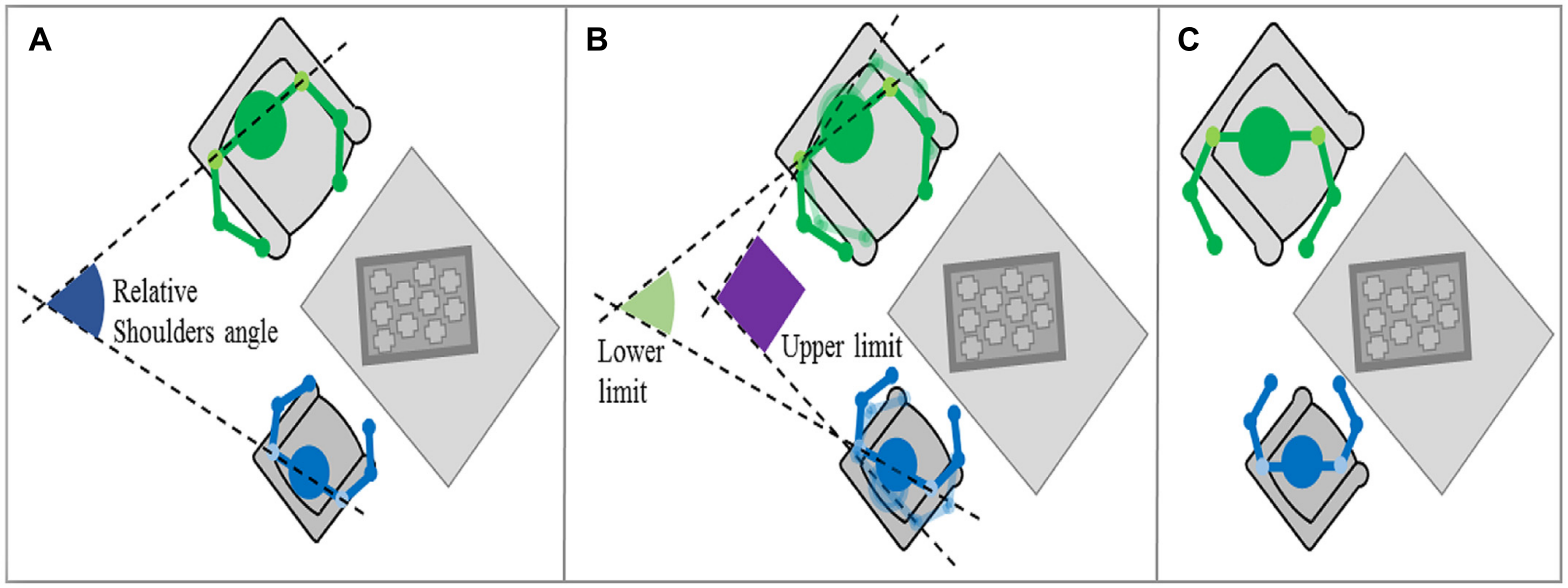
**Relative shoulder orientation feature. (A)** General case; **(B)** Same point of attention case; **(C)** Face to face case – top view representation.

**FIGURE 6:**
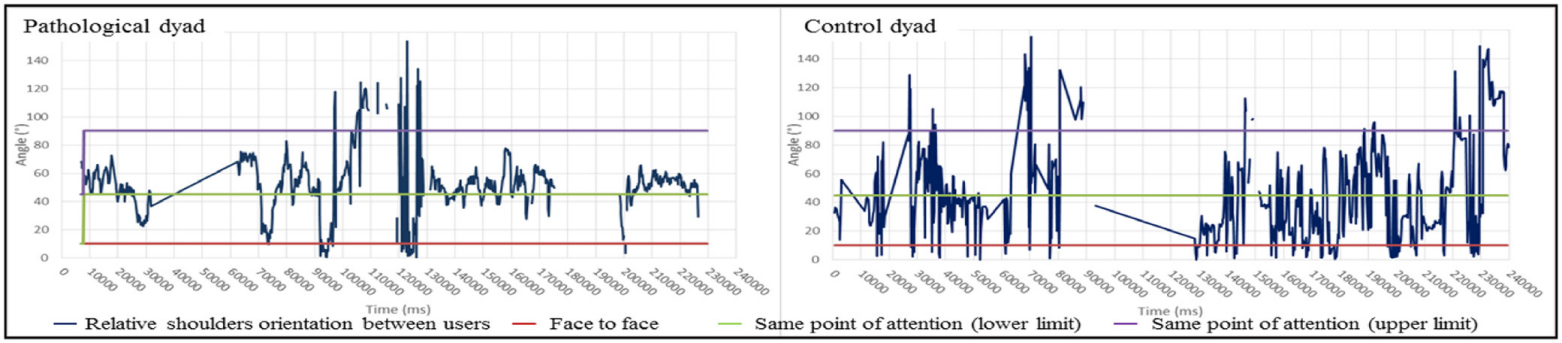
**Evolution of relative shoulder orientation during the interaction (Left: pathological dyad; Right: control dyad).** In this graph, we report the shoulder orientation according to the relative angle between the two partners’ shoulders over time. When the angle is equal to 0°, the partners are facing. When to the angle is 45 to 90°, both shoulders are oriented in the direction of the table that is a point of interest in the given task. In the left graph, the pathological dyad is focused essentially on the task, as partners are facing only three times. In contrast, the control dyad had many face to face positions and showed clear turns between task focusing and other partner focusing. A blank or a cross line in figures indicates uncollected data.

It is possible to determine the relative orientation between two persons using the same method used for the shoulder orientation. This was defined as the angle between the line formed by the parent’s shoulders and the child’s shoulders (see **Figure 5** for a graphical representation). Therefore, if parent and child are face to face, the angle will be close to 0° (red line in graphs, **Figure 5C**), while if they are facing the same area, the angle will be oscillate between 45 and 90° (green and purple lines in graphs, **Figure 5B**). The results for this feature are available in **Figure 6**. The interpretations are summarized in **Table 3**.

**Table 3:** Attention to the task and the partner features – results analysis.

	Pathological dyad	Control dyad
Shoulder orientation	Mother mostly oriented toward table and bench Child focused almost exclusively on the table	Parent moves between table, bench and his child Child moves a lot, focuses on parent, table and bench
Relative shoulder orientation	Dyad focused essentially on the task, just three periods when they are facing	Many face to face interactions Dyad oscillates between task focusing and other partner focusing

Conclusion	Shoulder orientation of the child and parent in the pathological dyad is less mobile than the control dyad. That could be interpreted as a poorer ability to share attention while alternating the focus of attention. The control dyad illustrates a fluid alternation of attention

### PARTICIPATION IN THE TASK

In this section, we present higher level features related to synchrony and engagement during the interaction. First, as the shoulder center distance to the table center captures the attention to the task, the hand distance to the table center can express the involvement in the task. Second, by combining distance or audio features with motion energy or speaker localization, we assume that we assessed partner engagement during the interaction.

#### Hand distance to the table center results

**FIGURE 7:**
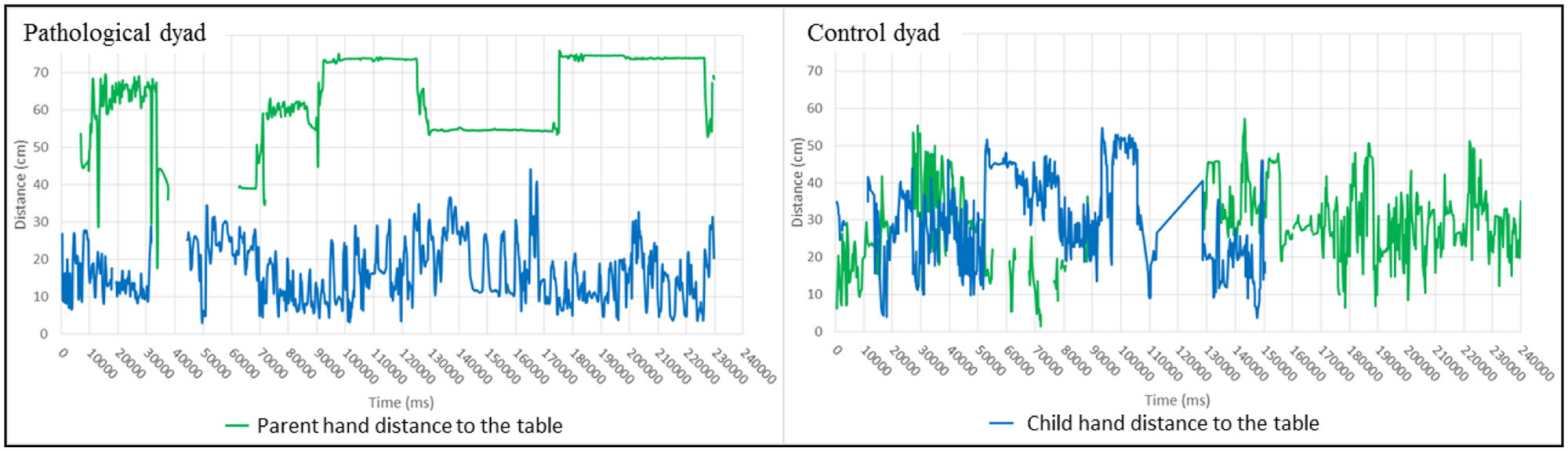
**Evolution of the distance between the closest hand and the table center during the interaction (Left: pathological dyad; Right: control dyad).** A blank or a cross line in figures indicates uncollected data. By convention, results concerning parents are in green, and results concerning children are in blue.

As explained above, the shoulder center distance to the table center captures the attention to the task because the hand distance to the table center can express the involvement in the task. If hands are close to the table, we can assume that the person is playing and therefore involved in the task. Unlike the shoulder centers distance feature, it is not the distance between the centers of the two hands that is studied, but the distance between the closest hand and the center of the gaming table (see **Figure 3A** for a top view representation of the feature). **Figure 7** shows the results for this feature. In the pathological dyad, only the child’s hand was close to the table and showed much activity. In contrast, in the control dyad, both the parent’s and child’s hands were close to the table and showed much activity.

#### Contribution to global movement

Contribution to the movement determines which partner participates in the global movement, and by studying the distance variations, it is possible to extract the type of movement in which the partner participates (avoidance or approaching). The objective of this feature is to detect when a movement is performed and who initiates it. In other words, if we look only at changes of the distance between the hands of the dyad (**Figure 4B**), we can see that there is some hand activity, but we cannot tell if the variation is due to movement of the parent or the child. To assess who engaged in changes in hand, head or torso distances, we defined a new parameter labeled contribution to the movement. When the distance between two points is tracked, the contribution is defined as the ratio between the velocity amplitude of one point and the sum of the velocity amplitudes of the two points.

**FIGURE 8:**
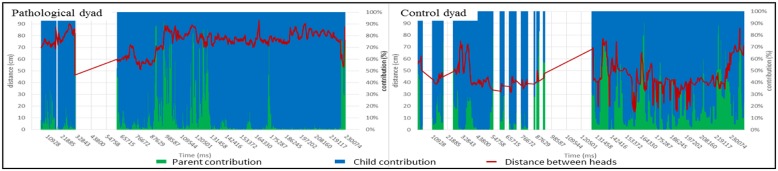
**Evolution of the distance between parent and child heads with each partner’s contribution to the global hand movement during the interaction (Left: pathological dyad; Right: control dyad).** In this graph, we report the distance between the parent’s and child’s heads with each partner’s contribution to the global hand movement during the interaction over time. At the same time, we are able to follow how close or distant partners are and who is moving the most in the previous frames, in other words, who is contributing the most to changing the distance. On the left graph, the pathological dyad showed a large head distance (minimum distance = 50 cm). Movements were initiated mostly by the child, except on two occasions. In contrast, the control dyad showed a smaller head distance (maximum distance = 75 cm). Movement contribution was distributed between the parent and child and the rhythm of the interaction appeared to be a motor dialog with many turns during the course of the interaction. A blank or a cross line in figures indicates uncollected data. By convention, results concerning parents are in green, and results concerning children are in blue.

This parameter has been computed with the distance between the parent and child heads feature. The results are presented **Figure 8**. At a given time, if the column is completely blue, it means that the current movement is due to the child, and conversely, if it is totally green, the parent is responsible for the movement. Moreover, if the distance (red line) increases, it means that the parent and child move away from each other, and if the distance decreases, they are approaching each other. **Figure 8** shows that in the pathological dyad, the heads were far apart and the child was the leader of the interaction. In contrast, in the control dyad, the heads were close and both the parent and child were the leaders of the changes during interaction, resulting in a motor dialog or movement turn taking. A detailed interpretation of this feature is given in the caption of **Figure 8**.

#### Sound activity associated with a participant

**FIGURE 9:**
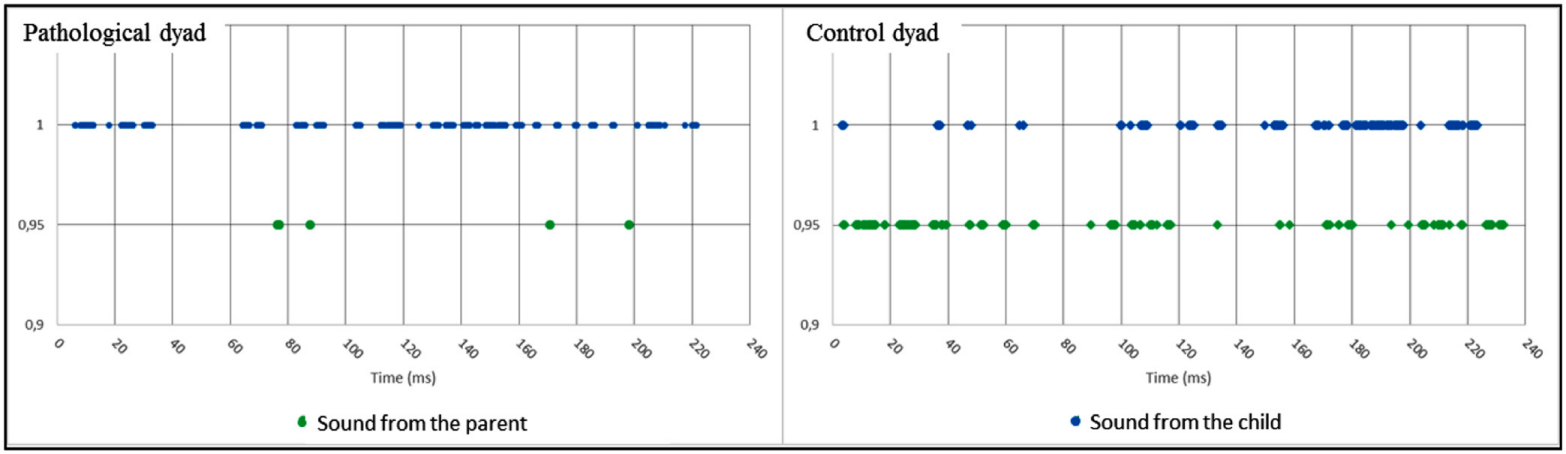
**Sound activity by participant during the interaction (Left: pathological dyad; Right: control dyad).** In this graph, we combined sound activity and source localization and report sound activity by participant during the interaction over time. In the left graph, the pathological dyad showed a clear disequilibrium. The majority of the sounds were produced by the child. The mother nearly always stayed silent. The dyad only had four speech turns during the entire interaction. In contrast, the control dyad showed no disequilibrium. Sounds were due equally to the child and the parent. Additionally, as in the motor analysis (see Figure), the rhythm resembled a dialog with numerous speech turns. By convention, results concerning parents are in green, and results concerning children are in blue.

The sound activity associated with a participant is a feature that parallels the visual modality in the contribution to global movement feature that we described above. In this feature, we combined audio activity with source localization that, in the context of the 3D-reconstruction, determines the speaker. **Figure 9** shows the results and a detailed analysis in the caption. In the pathological dyad, the majority of the sounds were due to the child. In contrast, in the control dyad, both partners contributed to the sound activity, and most importantly, many speech turns occurred, leading to an audio dialog.

## DISCUSSION

### SUMMARY OF THE RESULTS AND CROSS CORRELATION

We have developed an explorative method to acquire and extract relevant social signals from a naturalistic early parent–infant interaction in a clinical setting. We have extracted various cues from body postures and speech productions of each partner using the IMI2S Framework. Preliminary clinical and computational results for two dyads (one pathological in a situation of severe emotional neglect and one normal control) show that the absence of such interactive social signals indicates behavioral patterns that might be pathologically relevant: the pathological dyad shows dyssynchronic interaction led by the infant whereas the control dyad shows synchronic interaction and a smooth interactive dialog.

The goal oriented aspects (i.e., solving the task) are not affected whereas both the clinical assessment (CIB; **Figure 2**) and the computational feature extraction have revealed clear differences between the pathological and control dyads concerning the body/movement and sound activities of the parent and their involvement in the task and regarding the proximity and joint activity in the dyad. In other words, we can distinguish these two components and provide objective measures for when and how social communication is affected. The pathological parent avoided the activity and the child. This could be interpreted as avoidance of an interaction (Viaux-Savelon et al., 2014), meaning that the parent is less involved in the task and appears to be withdrawn. In contrast, the control dyad was characterized by a clearly distinguishable different dynamic: (1) distances between partners were mediated by movements toward and away from the partner in both the parent and child and (2) the number and regularity of speech turns was high, as in a dialog. These characteristics result in an illustration of synchrony and engagement switching during harmonious interactions (Delaherche et al., 2012).

### ATTENTION TO THE TASK AND THE PARTNER

Here, we present higher-level features related to each partner’s attention during the task and whether attention is oriented to the task or to the partner. These features are based on the assumption that if a person’s torso faces an area, the person’s attention is focused on this area. For example, if the parent’s chest is parallel to the table, it indicates that the parent is interested in the action occurring on the table. With the 3D reconstruction from the skeleton features, it was possible to determine the attention of the dyad to the gaming task and the parent’s attention to their child and *vice versa* by measuring each partner’s shoulder orientation and the relative shoulder orientation during the interaction.

The clinical assessment and the computational features do not share the same time scale. By this we mean that the CIB provides a summary of the whole interaction whereas the IMI2S data provides a much a more fine grained scale of the temporal flow. However, we propose the following cross correlation: (i) The “Parental Sensitivity” score of the CIB shows that the parent neglected his child and focused almost entirely on the task in the pathological dyad. CIB “Parental sensitivity” score may be associated with the parent’s shoulder distance to the table and the distance between the hands. Indeed, this clinical characteristic could be interpreted as the parent’s capacity to remain engaged in the interaction with a proximity adapted to child’s movements. (ii) The “Dyadic Reciprocity” score of the CIB clearly distinguishes the two dyads (not much enthusiasm, common involvement, reciprocal affection in the pathological dyad). By definition, a harmonious dyadic reciprocity means smooth and synchronous interaction entailing coordination between partners and intermodality (Feldman, 2007). CIB “Dyadic Reciprocity” may be related to the partners’ contributions to movement or speech turns that are equally distributed (**Figures 8** and **9**). (iii) Joint attention (a key item of the CIB “child’s engagement” score) can be illustrated by shoulder orientation and relative shoulder orientation (Anzalone et al., 2014a,b). For instance, a parent whose shoulders are oriented toward the same point for a majority of the time (see the pathological dyad in **Figures 4C** and **6**) can reveal a lack of adaptation to the child, preventing the occurrence of joint attention. In contrast, the control dyad showed a large variation of shoulder orientation, which can predict a good adjustment of attention between partners and shared attention (meaning attention of both partners toward a common object) during interactions.

In conclusion, for the current two case reports, computational feature extraction seems to provide the same results as clinical analysis, but allows a finer understanding of interactions by changing the time scale (from a summary of the whole interaction toward a more fine grained scale of the temporal flow) and by providing quantitative features that may be used in large comparison group data or single case longitudinal studies.

### LIMITATIONS

Even if the conclusions presented above are promising, the current results are subject to some limitations. First, given the exploratory nature of this study, any generalization of the findings is prevented; only two case-studies are compared, and even if they are paradigmatic, they cannot be statistically relevant and no statistics was applied. Second, the two cases were not matched for age or gender of the interactive parents but were chosen for their extreme PIRGAS scores. Third, at a group comparison level, it is likely that each pathological dyad would present different patterns of dyssynchrony such as intrusive or under involved styles. In this study, our pathological case was under-involved. Finally, extracted features (skeleton and audio) do not include every facet of the interaction (e.g., motherese). As a consequence, they could not be matched with all the subscores of the CIB.

### FUTURE STUDIES

This exploratory study encourages us to pursuing the study of the presented methodology and experimentation in new scenarios. This first work with these two dyads permits us to develop relevant sensor features in a clinical setting and a computational extraction system that can now be tested on a larger population. The next goal will be to accomplish a complete and statistically relevant comparison between the two groups by collecting data from a relevant number of dyads. In our future work, we will be specifically exploring intrusive or under involved parenting because the clinical validity should be tested in these different pathological patterns. We believe that the two features called “evolution of the distance between parent and child heads with each partner’s contribution to the global hand movement during the interaction” (**Figure 8**) and “sound activity by participant during the interaction” (**Figure 9**) will be clinically relevant at a group comparison level offering quantitative metrics for under involved parenting. Exploiting low-level signal exchanges allows proposing quantitative metrics without imposing meanings on the signals, which could be not only difficult but also limitative in clinical settings. Various metrics could be investigated ranging from information-based to machine-learning based (Delaherche et al., 2012). Possible metrics could be measuring entropy of individual activities (both infant and caregiver) for individual behavior characterization, mutual information between these activities for inter-personal synchrony characterization. We expect low values of synchrony metrics in pathological dyads whereas it should be higher in harmonious control dyads.

Furthermore, to complete the computational analysis, new features will be implemented in the IMI2S Framework. For example, the video stream recorded with the RGB-D sensor will be analyzed to extract the body activity of each participant or their head orientations (Anzalone et al., 2014a). Additionally, we will include a motherese classifier to better delineate parenting emotional prosody (Cohen et al., 2013). Our future hypothesis would be that these new features will confirm and improve the previous results. In particular, a combination of multimodal features will offer the ability to interpret and understand synchrony and dyssynchrony during early interactions in the context of neglected parenting (Glaser, 2002).

## Conflict of Interest Statement

The authors declare that the research was conducted in the absence of any commercial or financial relationships that could be construed as a potential conflict of interest.
